# Effect of sodium-glucose cotransporter-2 inhibitors on electrocardiographic markers of ventricular repolarization: A systematic review and meta-analysis

**DOI:** 10.1016/j.ahjo.2026.100888

**Published:** 2026-09-09

**Authors:** Muhammad Ghifari Karsa, Viyola Rahma, Muhammad Iqhramullah, Muhammad Habiburrahman

**Affiliations:** a Postgraduate Program of Public Health, Universitas Muhammadiyah Aceh, Aceh, Indonesia; b Faculty of Medicine, Imperial College London, London, United Kingdom

**Keywords:** Arrhythmia, Electrical remodeling, Electrocardiography, SGLT2 inhibitors, Ventricular repolarization

## Abstract

**Background:**

Sodium-glucose cotransporter-2 (SGLT2) inhibitors reduce sudden cardiac death and ventricular arrhythmias in cardiovascular outcome trials, but the electrophysiological mechanism remains unclear. Surface electrocardiographic (ECG) markers of repolarization dispersion may reflect myocardial electrical stability and provide mechanistic insight.

**Aim:**

To assess the impact of SGLT2 inhibitors on electrocardiographic markers of ventricular repolarization dispersion and conduction, and to evaluate whether these changes are consistent with improved ventricular electrical stability.

**Methods:**

We conducted a systematic review and meta-analysis of studies reporting ECG parameters before and after SGLT2 inhibitors and studies comparing SGLT2 inhibitors with other treatments. Searches were performed from database inception through June 2025 across Scopus, Web of Science, PubMed, Scilit, and Google Scholar. Random-effects inverse-variance models using restricted maximum likelihood with Hartung–Knapp adjustment were applied. Prespecified subgroup analyses were performed by follow-up duration.

**Results:**

Seventeen studies involving 5022 patients were included. In pre–post analyses, SGLT2 inhibitors significantly reduced QTc (MD −4.9 ms), Tp-e (MD −6.12 ms), P dispersion (MD −3.28 ms), and QTc dispersion (MD −1.36 ms), while R–R interval, PR interval, and QRS duration remained unchanged. Improvements in Tp-e-related indices and QRS–T angle were more pronounced at 6 months. In two-arm comparisons, SGLT2 inhibitors significantly shortened QT interval, QTc, Tp-e, Tp-e/QT, and Tp-e/QTc compared with controls.

**Conclusion:**

SGLT2 inhibitors reduce ECG markers of ventricular repolarization dispersion without affecting heart rate or conduction intervals, indicating improved ventricular electrical homogeneity.

## 1 Introduction

Sudden cardiac death (SCD) and ventricular arrhythmias remain major contributors to global mortality in patients with cardiometabolic disease, accounting for approximately 15–20% of all deaths worldwide [1], [2]. While many treatments improve hemodynamics or neurohormonal balance, fewer are known to directly influence the electrophysiological substrate that predisposes to malignant ventricular arrhythmias. Ventricular arrhythmogenesis is driven not only by structural abnormalities but also by heterogeneity of ventricular repolarization, commonly referred to as transmural and spatial dispersion of repolarization [3], [4].

Surface electrocardiographic markers such as QTc, Tp-e interval, Tp-e/QT, Tp-e/QTc, QT dispersion, QTc dispersion, and QRS–T angle have been shown to reflect this repolarization heterogeneity and are strongly associated with ventricular arrhythmias and cardiovascular mortality [5], [6], [7]. Increased Tp-e and widened QRS–T angle, in particular, have been linked to higher risk of malignant ventricular arrhythmias and sudden death [8], [9]. In contrast, parameters such as heart rate, PR interval, and QRS duration primarily reflect sinus node activity and conduction pathways and are less indicative of ventricular electrical instability. Changes in repolarization dispersion markers therefore provide insight into myocardial electrical remodeling that may not be captured by conventional clinical measures [10].

Sodium-glucose cotransporter-2 (SGLT2) inhibitors have consistently demonstrated reductions in sudden cardiac death, ventricular arrhythmias, and heart failure hospitalization across large cardiovascular outcome trials [11], [12], [13]. For example, in the DAPA-HF trial, dapagliflozin significantly reduced the composite risk of worsening heart failure and cardiovascular death [14]. Similarly, the EMPEROR-Reduced trial demonstrated that empagliflozin reduced cardiovascular death and heart failure hospitalization, with consistent benefits across arrhythmia-related outcome [12]. Emerging evidence also suggests a reduction in ventricular arrhythmic events and sudden death independent of glycemic control [15], [16]. These benefits appear early and occur across diverse patient populations, including those without overt heart failure, suggesting mechanisms beyond conventional metabolic improvement [11], [12].

The growing interest in the electrophysiological effects of SGLT2 inhibitors also aligns with contemporary strategies for SCD prevention. The 2023 Lancet Commission on Sudden Cardiac Death emphasized the importance of therapies that modify the arrhythmogenic substrate in addition to conventional approaches focused on risk stratification and device therapy. Emerging evidence suggests that SGLT2 inhibitors may influence several pathways involved in arrhythmogenesis, including myocardial energetics, intracellular sodium and calcium homeostasis, oxidative stress, fibrosis, and ventricular electrical remodeling. Understanding their effects on electrocardiographic markers of ventricular repolarization may therefore provide important mechanistic insights into their potential role in reducing ventricular arrhythmias and sudden cardiac death [17].

The electrophysiological mechanisms underlying these anti-arrhythmic effects remain incompletely understood. Proposed explanations include improvements in myocardial energetics, reduction in oxidative stress, attenuation of fibrosis, and improved microvascular perfusion, all of which can influence ventricular repolarization gradients [18], [19], [20]. Each of these mechanisms has the potential to reduce heterogeneity of action potential duration and transmural repolarization gradients, thereby stabilizing ventricular electrical activity [20]. Clarifying this distinction is important because dispersion-based ECG markers are mechanistically linked to arrhythmogenic substrate formation and may serve as practical indicators of myocardial electrical remodeling [4], [6].

Several clinical studies have reported changes in ECG parameters following SGLT2 inhibitors, but findings have been heterogeneous and have not been synthesized systematically. Moreover, it is unclear whether these changes primarily affect heart rate and conduction or selectively modify ventricular repolarization dispersion. This study aimed to evaluate the effects of SGLT2 inhibitors on ECG markers of ventricular repolarization and conduction. The review seeks to clarify whether SGLT2 inhibitors exert measurable effects on myocardial electrical stability and to provide mechanistic insight linking ECG changes with observed reductions in ventricular arrhythmic events.

Although SGLT2 inhibitors have been associated with reductions in ventricular arrhythmias and sudden cardiac death in major cardiovascular outcome trials, the electrophysiological correlates underlying these benefits remain incompletely characterized. Previous studies evaluating ECG-derived repolarization markers have reported heterogeneous findings across different cardiometabolic populations, and no prior synthesis has comprehensively evaluated whether these changes consistently reflect alterations in ventricular repolarization profiles.

## 2 Methods

### 2.1 Study design and search strategy

This systematic review and meta-analysis was conducted in accordance with the Preferred Reporting Items for Systematic Reviews and Meta-Analyses (PRISMA) 2020 statement. A comprehensive literature search was performed from database inception through June 2025 in Scopus, Web of Science, PubMed, Scilit, and Google Scholar. The search strategy combined MeSH terms and free-text keywords using Boolean operators. Search terms included variations of: “SGLT2 inhibitor” OR “sodium–glucose cotransporter 2 inhibitor” OR empagliflozin OR dapagliflozin OR canagliflozin OR ertugliflozin OR bexagliflozin AND “electrocardiogram” OR “ECG” OR “QT interval” OR “QTc” OR “QT dispersion” OR “QTc dispersion” OR “Tp-e” OR “P dispersion” OR “QRS duration” OR “PR interval” OR “QRS–T angle” OR “cardiac repolarization” OR “arrhythmia” OR “ventricular arrhythmia” OR “atrial fibrillation”. The search strategy was adapted for each database. No minimum publication year or language restrictions were applied for the literature search. Studies published in any language were eligible for inclusion. For non-English articles, data extraction was translated using automated translation (Google Translate), followed by verification by the reviewers to ensure accuracy. The study protocol was prospectively registered in the Open Science Framework (OSF) with registration link https://osf.io/ug3he/overview?view_only=550f110ddd044c999b728346a80bea93.

### 2.2 Eligibility criteria

Eligibility criteria were defined using the PICO framework. The population included adult patients (≥18 years) receiving sodium–glucose cotransporter-2 (SGLT2) inhibitors therapy, with underlying cardiovascular disease, diabetes mellitus, or heart failure. The intervention comprised SGLT2 inhibitors, including dapagliflozin, empagliflozin, canagliflozin, ertugliflozin, and bexagliflozin. The comparison group consisted of placebo, active comparators without SGLT2 inhibitors, or pre-treatment baseline in pre–post study designs. The outcomes of interest included electrocardiographic parameters such as QT interval, corrected QT (QTc) interval, QT dispersion (QTd), QTc dispersion (QTcd), Tp-e interval, Tp-e/QT and Tp-e/QTc ratios, P-wave dispersion, QRS duration, PR interval, QRS–T angle, and heart rate. Eligible study designs included randomized controlled trials (RCTs), non-randomized controlled studies, and observational studies with comparator groups or pre-post measurements. Review, editorials, conference abstracts without full data, case reports, animal studies, and studies without extractable ECG data were excluded.

Because ventricular repolarization characteristics may differ substantially between patients without overt structural heart disease and those with established cardiovascular disease, the included populations were expected to represent a broad electrophysiological spectrum ranging from predominantly metabolic to overt structural and arrhythmogenic substrates. Therefore, pooled estimates were interpreted as reflecting overall directional changes across heterogeneous cardiometabolic populations rather than disease-specific electrophysiological effects.

### 2.3 Study selection

After downloading, citation data were imported into Rayyan.ai for screening and selection. Duplicate records were initially identified using automated detection algorithm. All potential duplicates were then manually reviewed by the reviewers and final removal decisions were made based on consensus. Two independent reviewers (M.G.K. and V.R.) screened titles and abstracts for eligibility. Full-text articles were subsequently assessed for inclusion. Disagreements were resolved through discussion and consensus or adjudication by third reviewer when necessary. The study selection process was documented using a PRISMA flow diagram.

### 2.4 Data extraction

Data were extracted independently by two reviewers (M.G.K. and V.R.) using a standardized data collection form. Extracted variables included: study characteristics (author, year, country, design), sample size and population characteristics, type, dosage, and duration of SGLT2 inhibitors, comparator characteristics, and ECG outcomes. When outcomes were reported as median, ranges and interquartile ranges, they were converted to means and standard deviations using validated statistical methods on https://www.math.hkbu.edu.hk [21], [22]. For pre-post designs, changes scores were calculated as post-treatment minus pre-treatment values. The standard deviation of changes was estimated assuming a pre-post correlation coefficient (r) of 0.5, consistent with methodological recommendations when the true correlation is unknown. To assess the robustness of this assumption, sensitivity analyses were conducted using alternative correlation coefficients (*r* = 0.25 and *r* = 0.75). The consistency of pooled effect estimates, statistical significance, and heterogeneity across these assumptions was evaluated.

### 2.5 Outcome

QT dispersion and QTc dispersion were included because they remain reported in several contemporary clinical electrocardiographic studies assessing ventricular repolarization heterogeneity, although their methodological limitations and reproducibility concerns have been recognized.

### 2.6 Risk of bias assessment

The risk of bias of included studies was assessed independently by two reviewers (M.G.K. and V.R.) using the ROBINS-I tool. Discrepancies were resolved through discussion or consultation with third reviewer. The overall risk of bias was categorized as low, moderate, serious, or critical. The result of the risk of bias assessment is presented in the Supplementary material.

### 2.7 Meta-analysis and statistical analysis

Continuous outcomes were synthesized as mean differences (MDs) with 95% confidence intervals (CIs) because all ECG parameters were reported on identical measurement scales (milliseconds or ratios). Study-level means, standard deviations (SDs), and sample sizes were extracted for intervention and comparator arms. For pre–post studies, mean change scores were calculated as post-intervention minus pre-intervention values. The standard deviation of the change was derived assuming a pre–post correlation coefficient of *r* = 0.5, in accordance with Cochrane recommendations. Sensitivity analyses using alternative pre-post correlation coefficients (*r* = 0.25 and *r* = 0.75) yielded effect estimates that were comparable to the primary analyses using *r* = 0.5. The direction and statistical significance of the principal outcomes remained unchanged, indicating that the findings were robust to different assumptions regarding the correlation between pre- and post-treatment measurements. When studies provided multiple comparisons sharing the same SGLT2 inhibitors arm (versus DPP4 inhibitors or versus sulfonylureas), the SGLT2i sample size was evenly divided across comparisons to avoid unit-of-analysis error while retaining the original means and SDs.

Pooled estimates were calculated using the inverse-variance method under a random-effects model. Between-study variance (τ^2^) was estimated using the restricted maximum likelihood (REML) approach. To provide more reliable inference in the presence of heterogeneity and small numbers of studies, the Hartung–Knapp adjustment was applied to compute confidence intervals and *p*-values for pooled effects. Statistical heterogeneity was assessed using Cochran's Q test and quantified using *I*^*2*^ and τ^2^, with confidence intervals for τ^2^ and τ derived using the Q-profile method. For outcomes exhibiting substantial between-study heterogeneity, 95% prediction intervals were additionally calculated to assess the range of effects that may be expected in future studies.

Prespecified subgroup analyses were conducted according to follow-up duration (≤3 months vs 6 months) to evaluate temporal differences in ECG changes. The direction of effect was defined such that negative MD values indicated shortening or reduction of the ECG parameter in the SGLT2 inhibitors group relative to control. All analyses were performed in R using the meta package. Pre-post analyses and controlled comparisons were evaluated separately because these designs provide different levels of causal inference. The pre-post analyses were intended to assess within-group temporal changes, whereas controlled comparisons were considered more appropriate for evaluating treatment-related effects.

### 2.8 Publication bias

Formal assessment of publication bias (such as funnel plot asymmetry or Egger's test) was not performed when fewer than 10 studies were available per outcome, consistent with Cochrane methodological recommendations due to limited statistical power in small meta-analyses.

### 2.9 Certainty of evidence

The certainty of evidence for each outcome was evaluated using the GRADE (Grading of Recommendations Assessment, Development and Evaluation) framework, considering risk of bias, inconsistency, indirectness, imprecision, and publication bias. The overall certainty was rated as high, moderate, low, or very low [23], [24]. The GRADE summary of findings is provided in the Supplementary material.

## 3 Results

### 3.1 Study selection

**Fig. 1:**
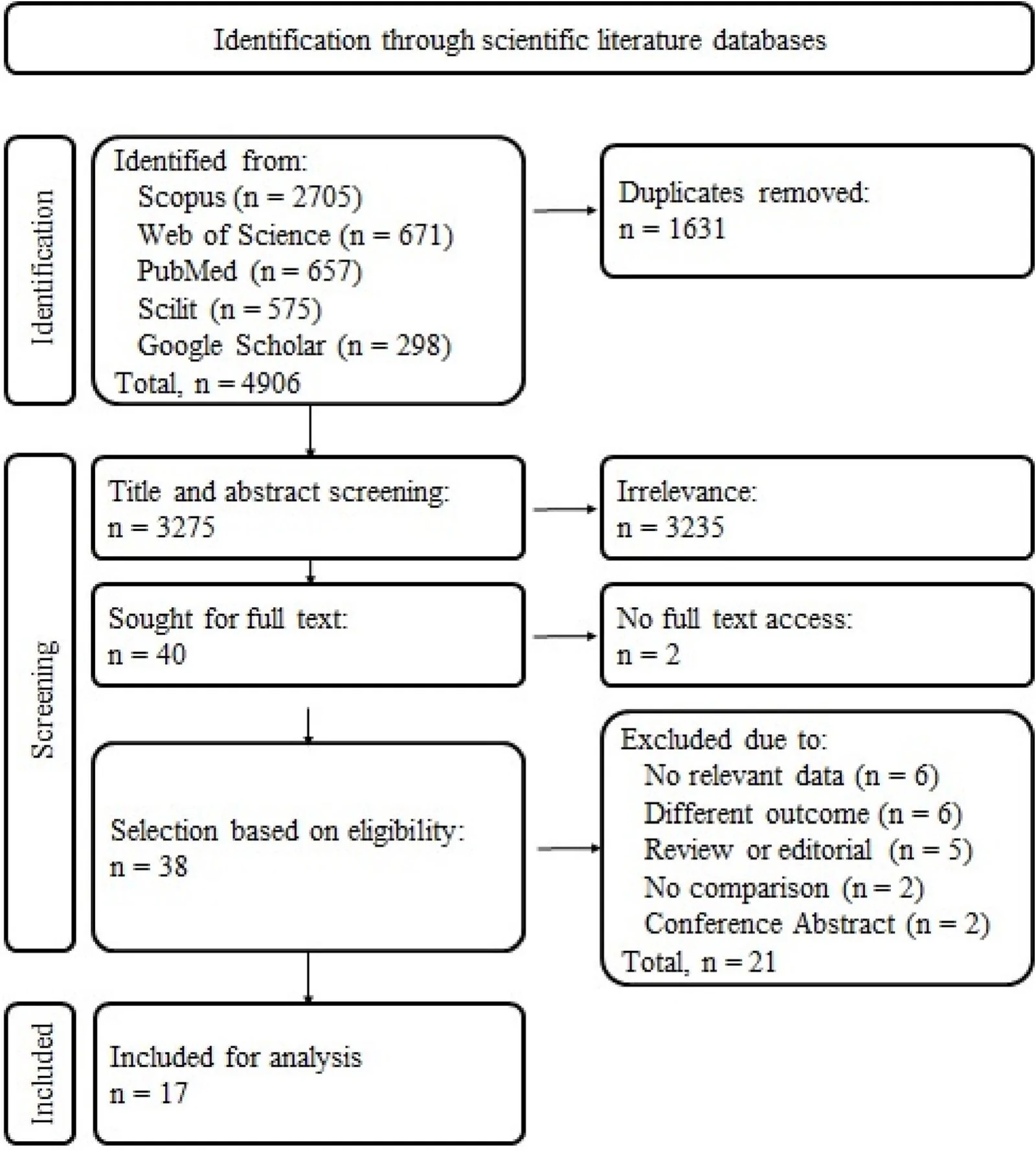
PRISMA flow diagram of study selection.

The literature search identified a total of 4906 records from five electronic databases, including Scopus (*n* = 2705), Web of Science (*n* = 671), PubMed (*n* = 657), Scilit (*n* = 575), and Google Scholar (*n* = 298). After removing 1631 duplicate records, 3275 studies remained for title and abstract screening. During the screening phase, 3235 studies were excluded due to irrelevance. The full texts of 40 potentially eligible studies were assessed for eligibility. Two articles were excluded because the full text was not accessible. Among the remaining 38 studies, 21 were excluded for the following reasons: absence of relevant ECG outcome data (*n* = 6), different primary outcomes (*n* = 6), review or editorial articles (*n* = 5), absence of a comparator or baseline measurement (*n* = 2), and conference abstracts without sufficient data (*n* = 2). Finally, 17 studies met the inclusion criteria and were included in the quantitative synthesis (meta-analysis) [25], [26], [27], [28], [29], [30], [31], [32], [33], [34], [35], [36], [37], [38], [39], [40], [41]. The summary of the literature selection process is presented in Fig. 1. The detailed characteristics of included studies are provided in Table 1.

**Table 1:** Characteristics of included studies.

Author, year	Country	Design	Population	Intervention vs comparator	Sample size (n)	Age (years)	Follow-up (months)
*Controlled studies*							
Akhan et al., 2021	Turkey	Retrospective	T2DM	Empagliflozin vs SU	64 vs 69	65–66	≥6
Campos et al., 2025	Brazil	RCT	T2DM	Dapagliflozin + GDMT vs GDMT	87 vs 87	∼71	3
Duran et al., 2020	Turkey	Retrospective	T2DM	SGLT2i vs DPP4i vs SU	40 vs 53 vs 48	60–63	3 &6
Li et al., 2024	China	Experimental	HFpEF + PAF	Dapagliflozin + standard vs standard	100 vs 100	57–59	3, 6, & 9
Ozdemir et al., 2024	Turkey	Prospective	T2DM	SGLT2i + metformin vs metformin	143 vs 174	42–43	6
Wu et al., 2022	Taiwan	Retrospective	T2DM	SGLT2i vs non-SGLT2i	1056 vs 2119	∼63	≥12
Zarei et al., 2024	Iran	RCT	CABG patients	Empagliflozin vs placebo	43 vs 39	56–57	Perioperative

*Pre-post studies*							
Abdellah et al., 2024	Egypt	Prospective	HFrEF	Dapagliflozin	156	∼57	12
Aslan et al., 2021	Turkey	Prospective	T2DM	Empagliflozin	53	∼55	3
Antwi-Amoabeng et al., 2022	USA	Retrospective	Adults on SGLT2i	Empagliflozin	101	∼66	12
Eyuboglu & Celik, 2022	Turkey	Prospective	T2DM	Empagliflozin	111	∼51	3 & 6
Gokalp & Ozbeyaz, 2023	Turkey	Retrospective	T2DM + HFpEF	SGLT2i	76	∼64	6
Kaya, 2024	Turkey	RCT	T2DM	Empagliflozin	70	∼56	6
Ozen et al., 2024	Turkey	Retrospective	HFrEF	SGLT2i	106	∼56	3
Sato et al., 2017	Japan	Retrospective	T2DM	SGLT2i	46	∼60	∼8
Takase B. et al., 2022	Japan	Retrospective	CAD + T2DM	Luseoglifozin	30	∼77	>12
Yilmaz et al., 2022	Turkey	Retrospective	HFrEF	SGLT2i	51	∼64	1

CABG, coronary artery bypass grafting; CAD, coronary artery disease; DPP4i, dipeptidyl peptidase 4 inhibitors; GDMT, guideline-directed medical therapy; HFpEF, heart failure with preserved ejection fraction; HFrEF, heart failure with reduced ejection fraction; PAF, paroxysmal atrial fibrillation; RCT, randomized controlled trial; SGLT2i, sodium-glucose co-transporter 2 inhibitors; SU, Sulfonylureas; T2DM, type-2 diabetes mellitus;

### 3.2 Risk of bias of included studies

Overall, most included studies were judged to have moderate to serious risk of bias, primarily driven by confounding and selection bias. Confounding was the most frequent source of bias, as many studies did not adequately adjust for baseline cardiovascular risk factor, concomitant medications, or differences in disease severity. Selection bias was also observed in several studies due to non-randomized designs and inclusion of clinically heterogeneous populations. Bias in classification of interventions and deviation from intended intervention was generally low, as SGLT2 inhibitors exposure was clearly defined and treatment adherence was relatively consistent across studies. Similarly, bias due to missing data was low in most studies.

Moderate risk of bias was observed in outcome measurement, as electrocardiographic parameters such as QTc and Tp-e intervals are susceptible to variability depending on lead selection, measurement techniques, and observer interpretation. Bias in selective reporting was considered low to moderate, with no clear evidence of systematic outcome reporting bias. No studies were judged to have critical risk of bias, supporting the overall suitability of the included evidence for quantitative synthesis. Randomized controlled trials demonstrated lower risk of bias compared with observational studies. These limitations contributed to downgrading the certainty of evidence in the GRADE assessment. Detailed domain-level assessments are presented in Supplementary Fig. S1.

### 3.3 Pre-post meta-analysis of ECG parameters

These analyses describe within-group changes over time and should be interpreted as supportive evidence because they do not account for secular trends, regression to the mean, or concurrent interventions. The pre–post effects of SGLT2 inhibitors treatment on ECG parameters is presented in Table 2.

**Table 2:** Summary of pre-post meta-analyses of electrocardiographic parameters following SGLT2 inhibitors.

ECG parameter	Study, n	Pooled MD (random, HK)	95% CI	*p*-Value	*I*^*2*^ (%)
R–R interval/heart rate (bpm)	7	−0.60	−3.80 to 2.59	0.660	94.5
P dispersion (ms)	5	−3.28	−6.27 to −0.29	0.038	88.6
PR interval (ms)	4	−3.38	−16.89 to 10.13	0.484	93.6
QT interval (ms)	4	−4.06	−14.44 to 6.32	0.302	74.7
QT dispersion (ms)	2	−3.57	−36.07 to 28.92	0.395	85.8
QTc (Bazett) (ms)	8	−4.19	−8.01 to −0.36	0.036	60.6
QTc dispersion (ms)	3	−1.36	−1.96 to −0.76	0.010	0
QRS duration (ms)	4	−0.39	−4.86 to 4.08	0.800	50.2
Tp-e interval (ms)	4	−6.12	−11.24 to −1.01	0.032	76.5
Tp-e/QT ratio	3	−0.026	−0.086 to 0.033	0.197	96.3
Tp-e/QTc ratio	3	−0.023	−0.052 to 0.006	0.075	69.9
QRS–T angle (°)	3	−8.30	−17.12 to 0.52	0.056	79.4

No significant change was observed in R–R interval/heart rate (MD −0.60, 95% CI −3.80 to 2.59) and PR interval (MD −3.38, 95% CI −16.89 to 10.13). P dispersion decreased overall (MD −3.28, 95% CI −6.27 to −0.29). Tp-e decreased overall (MD −6.12, 95% CI −11.24 to −1.01). Tp-e/QT did not decrease significantly overall. Tp-e/QTc did not decrease significantly overall. QRS–T angle showed a non-significant overall reduction (MD −8.30, 95% CI −17.12 to −0.52). Significant reduction for Tp-e, Tp-e/QT, Tp-e/QTC, and QRS-T angle were observed specifically in the 6-months subgroup analysis. Substantial heterogeneity was observed across most parameters, particularly for R–R interval (*I*^*2*^ = 94.5%), PR interval (*I*^*2*^ = 93.6%), QT dispersion (*I*^*2*^ = 85.8%), Tp-e/QT (*I*^*2*^ = 96.3%), Tp-e (*I*^*2*^ = 76.5%), and QRS-T angle (*I*^*2*^ = 79.4%), while QTc dispersion showed no heterogeneity (*I*^*2*^ = 0%). Some estimates were derived from only two or three studies and should be interpreted cautiously.

### 3.4 Two-arm meta-analysis of ECG parameters

**Fig. 2:**
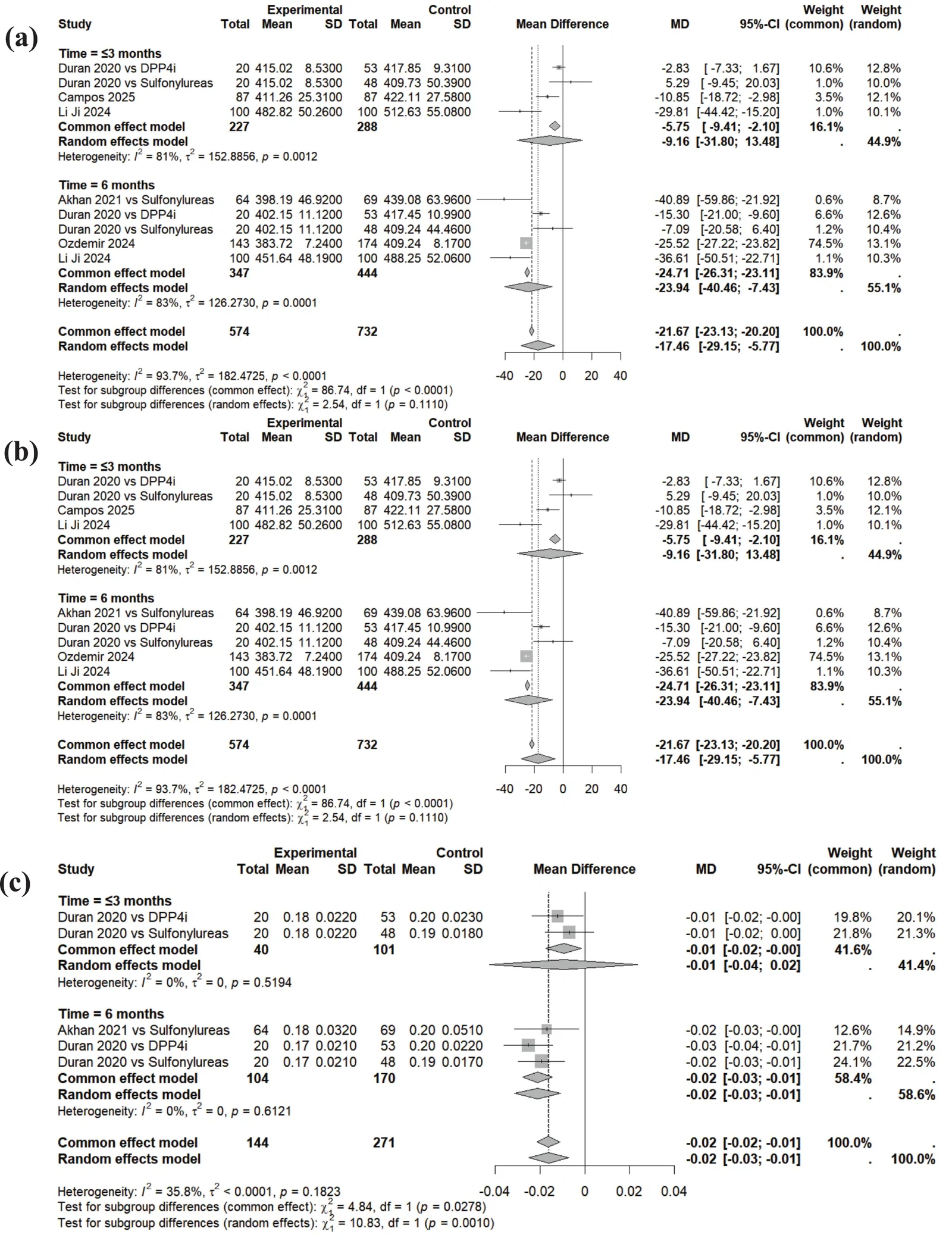
Forest plot of the effect of SGLT2 inhibitors compared with control therapies on corrected QT interval (a), T_peak_-T_end_ (Tp-e) interval (b), and Tp-e/QTc ratio.

The comparative effects of SGLT2 inhibitors versus control on ECG parameters are presented in Table 3. No significant difference was observed in R–R interval/heart rate (MD −2.30, 95% CI −6.07 to 1.47). QT interval decreased at 6 months (MD −12.32, 95% CI −14.49 to −10.16). QTc (Bazett) decreased at 6 months (MD −23.94, 95% CI −40.46 to −7.43). Tp-e decreased at ≤3 months (MD −6.40, 95% CI −8.57 to −4.23) and at 6 months (MD −11.64, 95% CI −22.92 to −0.36). Tp-e/QT decreased overall (MD −0.0165, 95% CI −0.0289 to −0.0041). Tp-e/QTc decreased overall (MD −0.0162, 95% CI −0.0251 to −0.0073). Considerable heterogeneity was observed for QT interval (*I*^*2*^ = 82.3%), QTc (*I*^*2*^ = 93.7%), and Tp-e/QT (*I*^*2*^ = 76.5%), while heterogeneity was moderate for R–R interval (*I*^*2*^ = 59%) and low for Tp-e (*I*^*2*^ = 39.6%) and Tp-e/QTc (*I*^*2*^ = 35.8%). Forest plot for the analysis presented in Fig. 2.

**Table 3:** Summary of two-arm meta-analysis comparing SGLT2 inhibitors with control treatments on electrocardiographic parameters.

ECG parameter	Study, n	Pooled MD (random, HK)	95% CI	*p*-Value	*I*^*2*^ (%)
R–R interval/heart rate (bpm)	5	−2.30	−6.07 to 1.47	0.165	59
QT interval (ms)	8	−7.81	−12.40 to −3.22	0.005	82.3
QTc (Bazett) (ms)	9	−17.46	−29.15 to −5.77	0.009	93.7
Tp-e interval (ms)	6	−8.67	−12.51 to −4.84	0.002	39.6
Tp-e/QT ratio	6	−0.0165	−0.0289 to −0.0041	0.019	76.5
Tp-e/QTc ratio	5	−0.0162	−0.0251 to −0.0073	0.007	35.8

### 3.5 Subgroup analysis by follow-up duration

Subgroup analyses were conducted according to follow-up duration, where the results are presented in Table 4.When stratified by follow-up duration, in subgroup analyses, studies with 6-month follow-up tended to report larger reductions in Tp-e, Tp-e/QT, Tp-e/QTc, and QRS-T angle than studies with shorter follow-up durations. However, these comparisons were conducted across different studies and should not be interpreted as evidence of a temporal or causal progression of treatment effects. At 6 months, significant improvements were observed in Tp-e (MD –9.00, 95% CI –13.07 to −4.93), Tp-e/QT ratio (MD –0.0500, 95% CI –0.0678 to −0.0322), and Tp-e/QTc ratio (MD –0.0400, 95% CI –0.0570 to −0.0230). In contrast, shorter follow-up periods (≤ 3 months) showed smaller or non-significant changes, while studies with ≥12 months demonstrated sustained reductions in repolarization indices.

**Table 4:** Subgroup meta-analysis of electrocardiographic outcomes according to follow-up duration.

ECG parameter	≤3 months MD (95% CI)	6 months MD (95% CI)	≥12 months MD (95% CI)	Overall MD (95% CI)	Heterogeneity		*p* (between groups)
					*I*^*2*^ (%)	*p*-Value	
R–R interval/heart rate (bpm)	0.69 (−47.28 to 48.65)	−1.00 (−3.37 to 1.37)	−1.43 (−6.86 to 3.99)	−0.60 (−3.80 to 2.59)	94.5	0.660	0.877
P dispersion (ms)	−3.75 (−7.79 to 0.29)	−1.50 (−2.66 to −0.34)	–	−3.28 (−6.27 to −0.29)	88.6	0.038	0.108
PR interval (ms)	−1.71 (−32.60 to 29.17)	–	−5.62 (−126.32 to 115.08)	−3.38 (−16.89 to 10.13)	93.6	0.484	0.69
QT interval (ms)	−0.71 (−57.82 to 56.41)	−6.80 (−13.09 to −0.51)	−13.00 (−25.59 to −0.41)	−4.06 (−14.44 to 6.32)	74.7	0.302	0.268
QT dispersion (ms)	−6.45 (−10.08 to −2.82)	−1.30 (−2.43 to −0.17)	–	−3.57 (−36.07 to 28.92)	85.8	0.395	0.0079
QTc (Bazett) (ms)	−4.99 (−6.73 to −3.25)	−6.20 (−14.43 to 2.03)	−2.32 (−12.67 to 8.02)	−4.19 (−8.01 to −0.36)	60.6	0.036	0.687
QTc dispersion (ms)	−0.96 (−4.92 to 3.00)	−1.40 (−2.50 to −0.30)	0.70 (−12.79 to 14.19)	−1.36 (−1.96 to −0.76)	0	0.010	0.935
QRS duration (ms)	−0.50 (−3.52 to 2.52)	0.00 (−3.49 to 3.49)	1.17 (−53.05 to 55.40)	−0.39 (−4.86 to 4.08)	50.2	0.800	0.926
Tp-e (ms)	−6.87 (−21.54 to 7.79)	−9.00 (−13.07 to −4.93)	−1.90 (−4.59 to 0.79)	−6.12 (−11.24 to −1.01)	76.5	0.032	0.0038
Tp-e/QT	−0.0158 (−0.1937 to 0.1620)	−0.0500 (−0.0678 to −0.0322)	–	−0.0263 (−0.0859 to 0.0332)	96.3	0.197	0.0408
Tp-e/QTc	−0.0178 (−0.0430 to 0.0075)	−0.0400 (−0.0570 to −0.0230)	–	−0.0232 (−0.0523 to 0.0058)	69.9	0.075	0.0123
QRS–T angle (°)	−6.01 (−28.94 to 16.92)	−11.50 (−14.45 to −8.55)	–	−8.30 (−17.12 to 0.52)	79.4	0.056	0.0194

### 3.6 Subgroup analysis of comparative studies

Subgroup analyses of two-arm studies showed consistent reductions across follow-up durations, presented in Table 5. At 6 months, significant reductions were observed in QT interval (MD –12.32, 95% CI –14.49 to −10.16), QTc (MD –23.94, 95% CI –40.46 to −7.43), and Tp-e interval (MD –11.64, 95% CI –22.92 to −0.36), with smaller effects observed at ≤3 months.

**Table 5:** Subgroup analysis of two-arm meta-analysis by follow-up duration.

ECG parameter	≤3 months MD (95% CI)	6 months MD (95% CI)	Overall MD (95% CI)	I2 (%)	p-Value	p (between groups)
R–R interval/heart rate (bpm)	−2.04 (−12.67 to 8.59)	−2.36 (−8.47 to 3.75)	−2.30 (−6.07 to 1.47)	59	0.165	0.899
QT interval (ms)	−2.47 (−5.94 to 1.01)	−12.32 (−14.49 to −10.16)	−7.81 (−12.40 to −3.22)	82.3	0.005	<0.0001
QTc (Bazett) (ms)	−9.16 (−31.80 to 13.48)	−23.94 (−40.46 to −7.43)	−17.46 (−29.15 to −5.77)	93.7	0.0088	0.111
Tp-e (ms)	−6.40 (−8.57 to −4.23)	−11.64 (−22.92 to −0.36)	−8.67 (−12.51 to −4.84)	39.6	0.0021	0.0497
Tp-e/QT	−0.0077 (−0.0158 to 0.0004)	−0.0262 (−0.0431 to −0.0094)	−0.0165 (−0.0289 to −0.0041)	76.5	0.0187	<0.0001
Tp-e/QTc	−0.0095 (−0.0425 to 0.0235)	−0.0211 (−0.0313 to −0.0108)	−0.0162 (−0.0251 to −0.0073)	35.8	0.0072	0.001

### 3.7 Certainty of evidence

The certainty of evidence ranged from low to moderate across outcomes. Moderate certainty was observed for Tp-e interval and Tp-e/QTc ratio, while most other outcomes were rated as low certainty due to heterogeneity and study design limitations. Detailed GRADE assessments are presented in Supplementary Table S2.

## 4 Discussion

This present systematic review and meta-analysis identified favorable changes in several ECG markers among patients receiving SGLT2 inhibitors. While pre-post analyses demonstrated improvements in multiple ventricular repolarization parameters, controlled comparisons provided more robust evidence regarding treatment-associated effects. The present meta-analysis shows a consistent reduction in ECG markers reflecting ventricular repolarization dispersion with the use of SGLT2 inhibitors, while parameters related to heart rate and conduction remain unchanged. QTc, Tp-e, Tp-e/QT, Tp-e/QTc, QT dispersion, QTc dispersion, and QRS–T angle decreased in pooled analyses across different follow-up durations, whereas R–R interval, PR interval, and QRS duration did not show a stable direction of effect. These findings are in line with a previous report suggesting the effect of SGLT2 inhibitors on lowering the risk and recurrence of atrial fibrillation [42]. Further, the patterns found in the present study indicate that the intervention primarily influences ventricular repolarization homogeneity, rather than sinus node activity or atrioventricular and intraventricular conduction, as suggested by previous studies [27], [31], [39], [40].

The interpretation of these findings should consider the substantial clinical heterogeneity of the included populations. Studies included patients with type 2 diabetes without overt structural heart disease as well as populations with heart failure, coronary artery disease, and atrial arrhythmias [5], [7]. Accordingly, baseline ventricular repolarization abnormalities and arrhythmogenic substrates were likely different across studies. In patients without established cardiovascular disease, ECG changes may predominantly reflect physiological electrophysiological modulation, whereas in patients with structural or functional cardiac disease, the observed effects may represent modification of pre-existing pathological repolarization heterogeneity. Therefore, the pooled estimates should not be interpreted as uniform effects across all clinical phenotypes [43], [44], [45].

QTc and Tp-e showed the most consistent reductions across analyses. In pooled estimates, QTc decreased significantly (MD –4.19, 95% CI –8.01 to −0.36), while Tp-e was also significantly reduced (MD –6.12, 95% CI –11.24 to −1.01). QTc reflects total ventricular repolarization time, whereas Tp-e and its ratios represent transmural dispersion of repolarization (TDR) [46]. TDR is a key electrophysiological substrate for re-entrant ventricular arrhythmias. The simultaneous shortening of QTc, Tp-e, and Tp-e-derived ratios therefore suggests a reduction in heterogeneity of action potential duration across the ventricular wall [26], [29], [30], [38].

The improvement in QRS–T angle observed in the present study further supports the aforementioned interpretation. In subgroup analyses, QRS–T angle decreased significantly at 6 months (MD –11.50, 95% CI –14.45 to −8.55). Its narrowing, particularly at 6 months, indicates improved coupling between depolarization and repolarization processes [47], [48]. Together, these findings are consistent with favorable ventricular electrical remodeling [31], [36].

In the present study, no significant changes were observed in R–R interval (MD –0.60, 95% CI –3.80 to 2.59), PR interval (MD –3.38, 95% CI –16.89 to 10.13), and QRS duration (MD –0.39, 95% CI –4.86 to 4.08). These parameters primarily reflect pacemaker activity, atrioventricular nodal conduction, and His–Purkinje conduction. Their stability indicates that the intervention does not exert a chronotropic or dromotropic effect. Instead, the effect appears specific to the repolarization phase of the ventricular action potential, suggesting modulation at the level of myocardial ionic currents and cellular electrophysiological stability [28], [39], [41].

Oxidative stress disrupts delayed rectifier potassium currents (IKr, IKs), prolonging and destabilizing ventricular action potentials [18], [49]. Reduction in myocardial oxidative stress restores these currents, resulting in shorter and more uniform repolarization [49]. Improved myocardial energetics, particularly a shift toward more efficient substrate utilization, stabilizes ATP-dependent ion pumps and calcium handling, further reducing repolarization heterogeneity [19], [20]. In addition, attenuation of myocardial fibrosis removes electrical discontinuities that create regional repolarization gradients, while improved microvascular perfusion reduces subendocardial ischemia, a key contributor to transmural repolarization differences [20], [50]. A reduction in sympathetic overactivity without inducing bradycardia may also decrease repolarization dispersion while leaving heart rate unchanged [50]. Collectively, these mechanisms may contribute to reductions in transmural and spatial dispersion of ventricular repolarization and are biologically compatible with the ECG changes observed in the present analysis. [20]

The temporal pattern observed in the present study is consistent with progressive electrophysiological changes over time. Although the overall pooled estimates demonstrated significant reductions in Tp-e and QTc, subgroup analyses suggested that the magnitude of these effects increased with longer follow-up duration. Reductions in Tp-e (MD –9.00, 95% CI –13.07 to −4.93), Tp-e/QT (MD –0.0500, 95% CI –0.0678 to −0.0322), Tp-e/QTc (MD –0.0400, 95% CI –0.0570 to −0.0230), and QRS–T angle (MD –11.50, 95% CI –14.45 to −8.55) were more pronounced at 6 months compared with ≤3 months. Shorter follow-up periods generally showed smaller or non-significant changes, whereas longer follow-up demonstrated sustained improvements in repolarization indices. These findings may reflect progressive myocardial remodeling over time, potentially related to improvements in oxidative balance, reduction of fibrosis, and enhanced microvascular and metabolic function [35], [37], [40].

These electrophysiological changes provide a plausible link to clinical observations from large cardiovascular outcome trials, where reductions in SCD and ventricular arrhythmias have been reported. Ventricular arrhythmogenesis is more closely related to dispersion of repolarization than to absolute QT prolongation. By reducing QTc, Tp-e, dispersion indices, and QRS-T angle, SGLT2 inhibitors may attenuate the underlying arrhythmogenic substrate at the myocardial level. Surface ECG markers may reflect improvements in ventricular electrical stability, although direct mechanistic confirmation cannot be established from ECG-based studies alone [14], [51]. Although QT dispersion has historically been proposed as a marker of spatial repolarization heterogeneity, its reproducibility and methodological robustness remain debated. Accordingly, the findings related to QT dispersion in the present analysis should be interpreted cautiously and viewed as supportive rather than definitive electrophysiological markers [52]. However, these findings were derived from a limited number of studies and were accompanied by substantial heterogeneity. Consequently, these estimates should be considered exploratory and require confirmation in larger prospective investigations.

The electrophysiological effects of SGLT2 inhibitors may vary according to the underlying clinical condition, particularly between patients with established cardiovascular disease and those without overt cardiovascular involvement. The included populations represented heterogeneous electrophysiological phenotypes, ranging from patients with relatively preserved cardiac structure to individuals with overt structural heart disease and arrhythmogenic substrates. However, the available studies included heterogeneous populations and generally did not provide outcome data stratified by disease status, limiting the ability to evaluate potential effect modification according to underlying cardiovascular disease. Future studies should investigate whether the effects of SGLT2 inhibitors on ventricular repolarization markers differ across specific clinical populations. Also, interpretation of these findings should consider the characteristics of the available evidence base. Most included studies were retrospective, single-center investigations with relatively small sample sizes, and a substantial proportion originated from a single country. These factors may limit the generalizability of the findings to broader populations and healthcare settings. In addition, several ECG markers evaluated in this review are subject to operator-dependent measurement variability and methodological differences across studies, which may have contributed to between-study heterogeneity. Thus, the present findings should be interpreted as electrophysiological associations derived from surface ECG markers rather than direct evidence of antiarrhythmic mechanisms.

Several other limitations should also be acknowledged. Most included studies were observational or retrospective, which introduces potential confounding and selection bias and limits causal inference, as reflected in the ROBINS-I assessment. Substantial heterogeneity was observed, likely driven by variations in sample size, ECG measurement methods, follow-up duration, and patient populations, including differences in underlying cardiometabolic conditions. Several ECG outcomes, including QT dispersion, QTc dispersion, Tp-e/QT, and Tp-e/QTc, were informed by only two to three studies. Consequently, estimates of between-study variance may be unstable and should be interpreted cautiously.

In addition, dispersion-based ECG parameters are inherently sensitive to methodological variability and interobserver differences. Accordingly, the overall certainty of evidence ranged from low to moderate based on the GRADE assessment. Furthermore, many included studies used single-arm pre-post designs, which are inherently susceptible to regression to the mean, secular trends, and co-intervention effects. Consequently, these findings should be interpreted as supportive rather than causal evidence regarding the electrophysiological effects of SGLT2 inhibitors. Despite these limitations, the consistent direction of effects across multiple repolarization parameters supports a biologically plausible impact of SGLT2 inhibitors on ventricular electrical remodeling.

## 5 Conclusion

SGLT2 inhibitors reduce ECG markers of ventricular repolarization dispersion, including QTc, Tp-e, Tp-e/QT, Tp-e/QTc, QT dispersion, QTc dispersion, and QRS–T angle, while no significant changes observed on heart rate and conduction intervals. These findings suggest potentially favorable effects of SGLT2 inhibitors on ventricular repolarization markers. However, the current evidence is largely derived from small observational studies and should be confirmed in larger multicenter prospective investigations using standardized ECG assessment methods. The findings from pre-post analyses should be interpreted as supportive evidence, whereas controlled comparative studies provide stronger evidence regarding treatment-associated electrophysiological effects. Future studies integrating ECG findings with electrophysiological mapping, ambulatory rhythm monitoring, cardiac imaging, and prospective arrhythmic outcomes are warranted. Large multicenter prospective studies are needed to validate these findings and further characterize the electrophysiological and structural correlates of these ECG changes.

## CRediT authorship contribution statement

**Muhammad Ghifari Karsa:** Writing – review & editing, Writing – original draft, Methodology, Investigation, Formal analysis, Data curation, Conceptualization. **Viyola Rahma:** Writing – review & editing, Writing – original draft, Methodology, Investigation, Formal analysis, Data curation, Conceptualization. **Muhammad Iqhramullah:** Writing – review & editing, Writing – original draft, Validation, Supervision, Formal analysis. **Muhammad Habiburrahman:** Writing – review & editing, Writing – original draft, Validation, Supervision, Funding acquisition.

## Ethical statement

This systematic review and meta-analysis was based on data extracted from previously published studies and did not involve direct contact with human participants, collection of new biological samples, or access to identifiable individual-level data. Therefore, ethical approval and informed consent were not required. The study protocol was prospectively registered in the Open Science Framework (OSF), and the review was conducted in accordance with the PRISMA 2020 statement.

## Declaration of competing interest

The authors declare that they have no known competing financial interests or personal relationships that could have appeared to influence the work reported in this paper.

## Appendix A Supplementary data

Supplementary material

Supplementary Table 2 GRADE summary of findings.
