# Supplementary material for: Effect of sodium-glucose cotransporter-2 inhibitors on electrocardiographic markers of ventricular repolarization: A systematic review and meta-analysis
Source: Am Heart J Plus. 2026 Sep 9;70:100888. doi: 10.1016/j.ahjo.2026.100888 (PMC13584065; doi:10.1016/j.ahjo.2026.100888)
Supplement: Supplementary file 1 [file mmc1.docx]

**Effect of Sodium-Glucose Cotransporter-2 Inhibitors on
Ventricular Repolarization and Electrocardiographic Dispersion:
A Systematic Review and Meta-analysis**

Muhammad Ghifari Karsa, Viyola Rahma, Muhammad Iqhramullah

**Risk of Bias and Reporting Sections for Systematic Review/Meta-analysis**

**1. Methods – Risk of Bias Assessment**

Risk of bias for each included study was assessed using the ROBINS-I tool. This instrument evaluates potential bias across seven domains:

(1) bias due to confounding,
(2) bias in selection of participants,
(3) bias in classification of interventions,
(4) bias due to deviations from intended interventions,
(5) bias due to missing data,
(6) bias in measurement of outcomes, and
(7) bias in selection of the reported result.

Two independent reviewers (M.G.K & V.R) evaluated each study following ROBINS-I guidance. Each domain was classified as low, moderate, serious, or critical risk of bias. Disagreements between reviewers were resolved through discussion until consensus was achieved. Because most included studies used observational or pre–post intervention designs, special attention was given to confounding factors such as baseline cardiovascular risk, concurrent medications, and follow‑up duration. The overall risk of bias judgement for each study was determined based on the highest level of bias observed in any domain. Results were visualized using traffic light plots and summary plots.

**2. Results – Risk of Bias**

Overall, most studies demonstrated moderate risk of bias, mainly driven by potential confounding and variability in ECG measurement methods. Bias related to classification of interventions, deviations from intended interventions, and missing data was generally low. Some studies showed moderate bias in outcome measurement because ECG parameters such as QTc and Tp‑e intervals can vary depending on lead selection, measurement technique, and observer interpretation. No studies were judged to have critical risk of bias,indicating that the overall evidence base remained suitable for quantitative synthesis.

**3. ROBINS-I Risk of Bias Table**

**4. Risk of Bias Summary**

| **Domain** | **Low** | **Moderate** | **Serious** |
| --- | --- | --- | --- |
| D1 | 2 | 10 | 5 |
| D2 | 2 | 14 | 1 |
| D3 | 10 | 7 | 0 |
| D4 | 17 | 0 | 0 |
| D5 | 14 | 3 | 0 |
| D6 | 0 | 17 | 0 |
| D7 | 0 | 17 | 0 |
| Overall | 2 | 10 | 5 |
