# Supplementary material for: Effect of sodium-glucose cotransporter-2 inhibitors on electrocardiographic markers of ventricular repolarization: A systematic review and meta-analysis
Source: Am Heart J Plus. 2026 Sep 9;70:100888. doi: 10.1016/j.ahjo.2026.100888 (PMC13584065; doi:10.1016/j.ahjo.2026.100888)
Supplement: Supplementary file 2 [file mmc2.docx]

**Supplementary Table 2. GRADE Summary of Findings**

| **Outcome** | **Study Design** | **Number of Studies (n)** | **Effect  (MD, 95% CI)** | **Risk of Bias** | **Inconsistency** | **Indirectness** | **Imprecision** | **Publication bias** | **Certainty of Evidence** | **Interpretation** |
| --- | --- | --- | --- | --- | --- | --- | --- | --- | --- | --- |
| QTc interval | Mixed (RCT + observational) | 9 | −17.46  (−29.15 to −5.77) | Serious | Serious (I²=93.7%) | Not serious | Not serious | Undetected | ⬤⬤◯◯ Low | May reduce QTc |
| QT interval | Mixed | 8 | −7.81  (−12.40 to −3.22) | Serious | Serious (I²=82.3%) | Not serious | Not serious | Undetected | ⬤⬤◯◯ Low | May shorten QT |
| Tp-e interval | Mixed | 6 | −8.67  (−12.51 to −4.84) | Serious | Not serious (I²=39.6%) | Not serious | Not serious | Undetected | ⬤⬤⬤◯ Moderate | Likely reduces TDR |
| Tp-e/QT ratio | Mixed | 6 | −0.0165  (−0.0289 to −0.0041) | Serious | Serious (I²=76.5%) | Not serious | Not serious | Undetected | ⬤⬤◯◯ Low | May improve dispersion |
| Tp-e/QTc ratio | Mixed | 5 | −0.0162  (−0.0251 to −0.0073) | Serious | Not serious (I²=35.8%) | Not serious | Not serious | Undetected | ⬤⬤⬤◯ Moderate | Likely improves dispersion |
| QTc dispersion | Observational | 3 | −1.36  (−1.96 to −0.76) | Serious | Not serious (I²=0%) | Not serious | Serious  (small n) | Undetected | ⬤⬤◯◯ Low | May reduce dispersion |
| P dispersion | Observational | 5 | −3.28  (−6.27 to −0.29) | Serious | Serious (I²=88.6%) | Not serious | Not serious | Undetected | ⬤⬤◯◯ Low | May reduce atrial dispersion |
| QRS duration | Observational | 4 | −0.39  (−4.86 to 4.08) | Serious | Moderate | Not serious | Serious  (CI crosses null) | Undetected | ⬤⬤◯◯ Low | No clear effect |
| PR interval | Observational | 4 | −3.38  (−16.89 to 10.13) | Serious | Serious (I²=93.6%) | Not serious | Serious | Undetected | ⬤⬤◯◯ Low | No clear effect |
| Heart rate (R–R) | Observational | 7 | −0.60  (−3.80 to 2.59) | Serious | Serious (I²=94.5%) | Not serious | Serious | Undetected | ⬤⬤◯◯ Low | No clear effect |
| QRS–T angle | Observational | 3 | −8.30  (−17.12 to 0.52) | Serious | Serious (I²=79.4%) | Not serious | Serious (borderline CI) | Undetected | ⬤⬤◯◯ Low | Uncertain effect |
